# Supplementary material for: Tobacco Plastid Transformation as Production Platform of Lytic Polysaccharide MonoOxygenase Auxiliary Enzymes
Source: Int J Mol Sci. 2022 Dec 24;24(1):309. doi: 10.3390/ijms24010309 (PMC9820616; doi:10.3390/ijms24010309)
Supplement: Supplementary file 1 [file ijms-24-00309-s001.zip › ijms-2089639-supplementary.pdf]

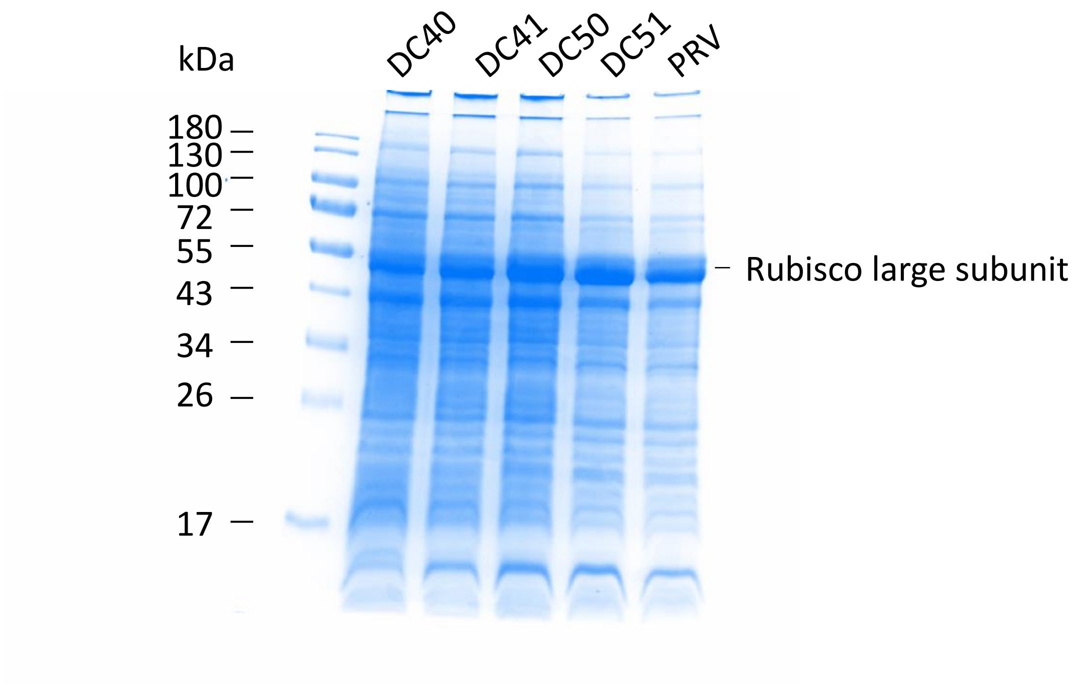

Supplementary Figure S1. Protein profile of transplastomic plants. Coomassie blue staining of polyacrylamide gel (original, uncropped and unadjusted image) of crude extracts from transplastomic plants expressing AA9 LPMO enzymes TaAA9B (DC40 and DC41) and TrAA9B (DC50 and DC51). The band corresponding to the Rubisco large subunit has been indicated. PRV, control plants transformed with an empty vector.
